# Chlorate of Potash Poisoning

**Published:** 1882-02

**Authors:** 


### Chlorate of Potash Poisoning.

The use of chlorate of potash as a household remedy, especially for children, is so common, that it is well to note the somewhat frequent occurrence of the fatal effect of overdoses of this drug. Dr. Satlow, of Leipzig, reports the case of a boy fifteen and a half years old, convalescent from diphtheria, who was attacked with symptoms of poisoning after swallowing a solution of chlorate of potash and water amounting to from twenty-five to thirty grammes of the salt. On the night of December 24th, after drinking the mixture, he was seized with frequent vomiting of dark green masses very similar to thin fæcal discharges; at midnight a small quantity of dark urine was passed; at daybreak the patient was noticed to be jaundiced. December 25th, 9 A. M., the temperature was 37° C.; pulse 124, weak; respirations 40. Skin cyanotic; lungs normal; heart sounds normal, excepting that the first sound was somewhat prolonged; some epigastric tenderness; liver enlarged and palpation both in this region and over the spleen, which was also enlarged, caused great pain. There was suppression of urine, none having been excreted since the small quantity passed in the night, the bladder having been found by the cathether to be empty. The patient complained of weakness, præcordial anxiety, and dyspnœa; the mind was clear; the vomiting continued every fifteen minutes. The anuria continued until December 26th, 4 A. M., when a few drops of dark-red, dense urine were passed accompanied by burning pain; the vomiting continued. Temperature 38.2° C.; pulse 104; respirations 28. At 4 P. M. the patient felt a little better, but a slight convergent strabismus of the left eye was noticed. The symptoms continued, although stimulants were freely given and transfusion resorted to twice, and on the morning of December 28th the patient died, his mind remaining clear to the last, and death resulting gradually from increased weakness of the heart, accompanied by dyspnœa and subjective feelings of coldness and paralysis of the feet progressively extending upwards. The post-mortem appearances, besides showing intense catarrh of the gastro-intestinal canal and enlargement of the liver and spleen, were especially interesting as showing the effect of the chlorate of potash on the blood, which was similar to the results obtained by the experiments of Marchand with this salt, the blood having the characteristic brown color (lackfarbig) and the density of syrup and the red corpuscles being especially affected, becoming pale and glutinous and gathering together in irregular clumps. A large quantity of reddish-brown fragments, supposed to be hæmoglobin, had been found in the urine passed on December 26th, and on examination of the kidneys these same masses were found in large numbers, especially in the convoluted and straight tubules, only sparingly in the glomeruli, and not at all in the interstitial tissue. It was also noticeable that there was no sign of an inflammatory condition anywhere in the kidney, the interstitial tissue being absolutely normal, and the epithelial cells of the tubules, although compressed by the masses of hæmoglobin, showing no trace of cloudiness or infiltration.—*Boston Med. and Surg. Jour.*

